# Recurrent chromosomal and epigenetic alterations in oral squamous cell carcinoma and its putative premalignant condition oral lichen planus

**DOI:** 10.1371/journal.pone.0215055

**Published:** 2019-04-09

**Authors:** Christopher G. Németh, Christoph Röcken, Reiner Siebert, Jörg Wiltfang, Ole Ammerpohl, Volker Gassling

**Affiliations:** 1 Department of Oral and Maxillofacial Surgery, University Hospital of Schleswig-Holstein, Kiel, Germany; 2 Department of Pathology, University Hospital of Schleswig-Holstein, Kiel, Germany; 3 Institute of Human Genetics, University Hospital of Schleswig-Holstein, Kiel, Germany; 4 Institute of Human Genetics, University Medical Centre, Ulm, Germany; Sapporo Ika Daigaku, JAPAN

## Abstract

Head and neck squamous cell carcinoma (HNSCC) affects about 700.000 individuals per year worldwide with oral squamous cell carcinoma (OSCC) as a major subcategory. Despite a comprehensive treatment concept including surgery, radiation, and chemotherapy the 5-year survival rate is still only about 50 percent. Chronic inflammation is one of the hallmarks of carcinogenesis. Until now, little is known about the premalignant status of oral lichen planus (OLP) and molecular alterations in OLP are still poorly characterized. Our study aims to delineate differential DNA methylation patterns in OLP, OSCC, and normal oral mucosa.

By applying a bead chip approach, we identified altered chromosomal patterns characteristic for OSCC while finding no recurrent alterations in OLP. In contrast, we identified numerous alterations in the DNA methylation pattern in OLP, as compared to normal controls, that were also present in OSCC. Our data support the hypothesis that OLP is a precursor lesion of OSCC sharing multiple epigenetic alterations with OSCC.

## Introduction

Head and neck squamous cell carcinoma (HNSCC affects about 700,000 individuals per year currently making it the sixth leading cause of cancer-related mortality worldwide [1, 2]. Among these, HNSCC of the lip, oral cavity and pharynx have been estimated to account for 529,500 incident cases and 292,300 deaths in 2012 [2]. Despite a comprehensive treatment concept including surgery, radiation, and chemotherapy the 5-year survival rate of patients carrying these tumours is still only about 50 percent [2]. Thus, further insight into HNSCC pathogenesis is urgently needed to improve preventive and therapeutic strategies.

The development of HNSCC is a multistep process characterised by the accumulation of genetic and epigenetic alterations leading to activation of oncogenes and inactivation or loss of tumour suppressor genes [3], including frequent DNA copy number gains at chromosomes 3q, 5p and 8q, as well as copy number losses on 3p and 8p [4]. To date, several driver genes in HNSCC like *TP53*, *CDKN2A*, *PIK3CA*, *HRAS*, and *FBXW7* have been identified [5].

Besides genetic alterations, virtually all cancers are associated with aberrant DNA methylation, in particular, a genome-wide hypomethylation of repetitive sequences and hypermethylation of high CpG-content promoters and target genes of the polycomb repressive complexes (PRC1 and PRC2) [6–15]. This results in genomic instability and contributes to altered gene expression and cell differentiation [16].

Locus-specific and global alterations of methylation have also been reported in HNSCC [17–20]. Recently, the global hypomethylation and gene-specific methylation processes have been observed in a series of 138 HNSCCs and the authors proposed that clinical characteristics and exposures influencing disease processes lead to HNSCC [21]. Furthermore, it has been shown that hypermethylation among 13 CpG loci, characterized by polycomb gene targets, mammalian interspersed repetitive elements and transcription factor binding sites was associated with reduced survival in patients with HNSCC [22]. A number of locus-specific studies have investigated the methylation status of certain tumour-suppressor genes. In this context, it has been proposed that the inactivation of p16 might play a crucial role in carcinogenesis of several cancers including HNSCC [23]. Concerning the development of oral cancer hypermethylation of p16^Ink4a^ and p14^ARF^ was detected in 57.7% respectively 3.8% of patients with oral epithelial dysplasia [24]. The highest rates of hypermethylation occurred in lesions of the tongue and mouth base. The hypermethylation of promoters of cancer-related genes in HNSCC has also been widely reported [25–28]. The wide range of frequencies observed in different studies was explained by varying environmental factors encountered by each population and distinct genetic pathways involved [29].

Chronic inflammation is known to be a crucial factor at the interface between genetics and the environment which may contribute to cancer development. Oral lichen planus (OLP), a chronic mucocutaneous autoimmune disease of unknown aetiology is still being discussed as a precursor lesion of OSCC, while controversial results do not allow for definitive conclusions [30–32]. In contrast to premalignant lesions, such as leukoplakia and erythroplakia, proof of molecular alterations in OLP is still lacking [33, 34] and data concerning methylation patterns in OLP are very limited. Recently, the investigation of prognostic biomarkers in OLP and oral squamous cell carcinoma (OSCC) showed that *p16[CDKN2A]* methylation and *miR-137* promoter methylation occur with a frequency of 25% and 35% respectively in patients with OLP, and 50% and 58.3% respectively, in patients with OSCC. In healthy subjects, however, *p16[CDKN2A]* methylation and *miR-137* promoter methylation were virtually absent [35]. A recently published study aimed to assess the methylation status of different candidate genes in OSCC, high-grade squamous intraepithelial lesions (HG-SIL), low-grade SIL (LG-SIL), OLP, and eight healthy donors in order to detect early OSCC and potential precursor lesions. The authors state that the aberrant DNA methylation of *GP1BB* and *ZAP70* represent promising tools for the early detection of OSCC and HG-SIL [36].

Our study aims to delineate differential DNA methylation patterns in OLP, OSCC and healthy tissue which might shed further light on the pathogenesis of OSCC and its potential precursor lesions.

## Materials and methods

### Patient recruitment

Patient recruitment and sample selection were described in detail previously [37]. Briefly, native tissue samples were collected at the Department of Oral and Maxillofacial Surgery, University Hospital of Schleswig-Holstein, Campus Kiel, Germany. Due to the strict inclusion criteria from initially 117 patients, eight with reticular OLP in their past medical history and typical clinical features were finally selected and specimens were taken at regular intervals. Diagnosis of OLP was verified by board-certified surgical pathologists of the Department of Pathology applying the specified histopathological criteria detailed previously [37]. Fifteen samples of OSCC were obtained during surgery and handled as described above to verify tumour diagnosis and to be stored for further analysis. These samples were taken from inside the main tumour mass but outside of obviously necrotic tissue and underwent a histopathological examination to provide the best possible homogeneity. The control group was composed of eighteen normal oral mucosa samples from elective oral surgery whose anamnestic data and histopathological examination excluded any diseases of the oral mucosa (Table 1 and S1 Table).

**Table 1:** Overview of the samples used in this study.

**group**	**n**	**ratio f:m**	**female (%)**	**male (%)**	**age (median, range)**	**smoking**	**alcohol**	**HPV**
**Normal control tissue**	18	13:5	72.2	27.8	25.5 (17–69)	8	9	0
**OLP**	8	6:2	75	25.0	60.5 (50–73)	4	6	0
**OSCC**	15	7:8	46.7	53.3	63.0 (43–89)	11	14	0

The study design complied with the Declaration of Helsinki and was approved by the ethics board of the Christian-Albrechts-University of Kiel, Germany (reference number: D 426/08). All patients gave written informed consent before being included in our study.

### Human papillomavirus status

All patient samples had to be negative for human papillomavirus (HPV) infection, which was tested as follows. DNA was extracted from formalin fixed and paraffin embedded tissue sections using the QIAamp DNA Mini Kit (Qiagen, Hilden) and then centrifuged for further purification. The isolated DNA was checked for its concentration and quality using the BIOMED-2-protocol and after verification was amplified via PCR. For this the primer sets MY09/11 and GP5^+^/6^+^ targeting the HPV-specific L1-region were used, having been tested for reliability and repeatability [38].

### Array-based DNA-methylation analysis

Array-based DNA-methylation analysis using the HumanMethylation450 BeadChip was performed as described in detail previously [39]. DNA was extracted from fresh frozen tissue using standard methods. To prevent a sex-related analysis bias, data of loci located on chromosomes X and Y were excluded. After quality filtering a total of 41 hybridizations, 471,336 loci entered the final analyses (Table 1 and S1 Table). Results are available in a MIAMI compliant format from Gene Expression Omnibus (GSE123781). Data were exported from the Genome Studio software and the Omics Explorer (ver.3.0 (25); Qlucore, Lund, Sweden) was used for further analyses. False discovery rate (FDR) and/or variance (σ/σ_max_) filters applied for individual analyses are given in the respective descriptions of the results. Microsoft Excel 2010 and R (R Core Team. R: A Language and Environment for Statistical. R Foundation for Statistical Computing, Vienna, Austria, https://www.R-project.org) were used for graphical presentation of the data.

### Analyses of chromosomal alterations

The R-package “CopyNumber450k” applying the standard settings on the HumanMethylation450 BeadChip data was used to analyse chromosomal alterations (https://bioconductor.riken.jp/packages/3.1/bioc/html/CopyNumber450k.html) in OLP, OSCC as well as control tissue samples. The control data set provided with the R-package acted as a reference for identifying genetic alterations (R-package “CopyNumber450kData”). P-values <0.01 as determined by the software package were considered as significant. Furthermore, chi-square statistics to calculate enrichment of particular characteristics as well as further statistic tests have been performed using R (R Core Team. R: A Language and Environment for Statistical. R Foundation for Statistical Computing, Vienna, Austria, https://www.R-project.org) as well as Prism software (ver. 4.02; Graph Pad Software, San Diego, CA).

### Analyses of the chromosomal state

Information on NHEK (normal human epidermal keratinocytes) cells provided by the Ensembl database (http://www.ensembl.org/info/website/news_by_topic.html?db=core; topic = regulation) was used to correlate each individual locus present on the HumanMethylation450k BeadChip with a unique chromosomal state in this cell type. Enrichment of aberrantly methylated loci in OLP or OSCC as compared to normal controls located in distinct regulatory regions was calculated. A list of all loci included in the differential methylation analysis acted as a reference list. Enrichments of loci located in CpG-islands or known differentially methylated regions (DMR) were calculated based on the information provided by Illumina with a list of all CpG-loci included into the study acting as background/reference list by performing a chi-square statistic.

### Calculation of methylation age

To calculate the epigenetic age of samples based on the DNA methylation pattern we used the DNAm age calculator provided at https://dnamage.genetics.ucla.edu/

### Gene ontology analysis

Gene ontology analysis for enrichment of biological processes, molecular function and protein binding among differentially methylated genes was performed using the *Gene Ontology enRIchmentanaLysis and visuaLizAtion tool* GOrilla, http://cbl-gorilla.cs.technion.ac.il/, accessed 01/2016 [40, 41].

## Results

### Recurrent chromosomal alterations in oral lichen planus and oral squamous cell carcinoma

**Fig 1:**
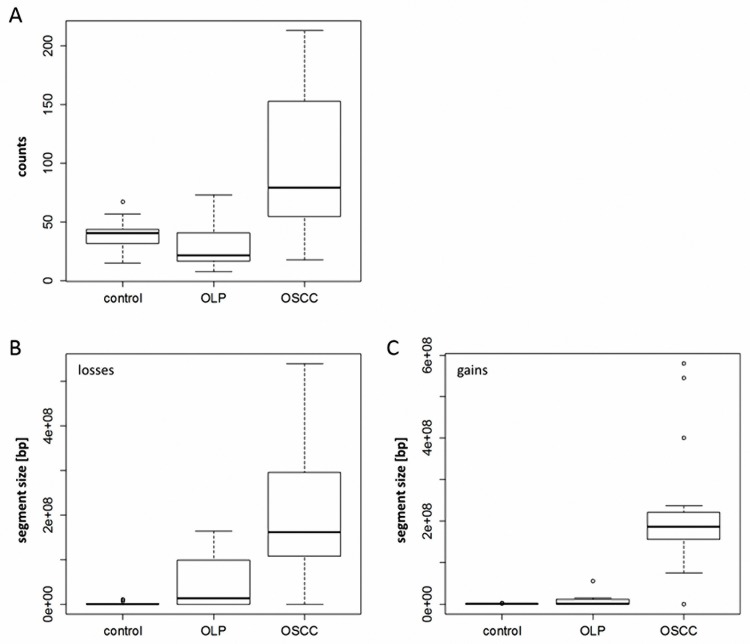
Chromosomal alterations in normal oral squamous cells (control), oral lichen planus (OLP) and oral squamous cell carcinoma (OSCC). The absolute number (counts) of detected alterations in the individual groups (A), as well as the size of losses (B) and gains (C), are presented as box plots indicating quartiles and medians.

In the first step, we analysed the number as well as the size of genetic copy number variations (CNV) in control tissue, OLP and OSCC for sequence gains and losses separately. For this, we applied the approach described by Feber and colleagues, who reported suitability of HumanMethylation450k BeadChip data for CNV analysis [42]. While no significant difference in either the number of CNVs between controls and OLPs or the sizes of gained or lost fragments could be detected, the number of CNVs observed in OSCC was significantly increased as compared to controls (p<0.01) and OLPs (p<0.01). Furthermore, the size of either gained (control vs. OSCC: p<0.00001; OLP vs. OSCC: p<0.001) or lost regions (control vs. OSCC: p<0.0001; OLP vs. OSCC p<0.01) was significantly increased in OSCC as compared to controls or OLPs (Fig 1).

**Fig 2:**
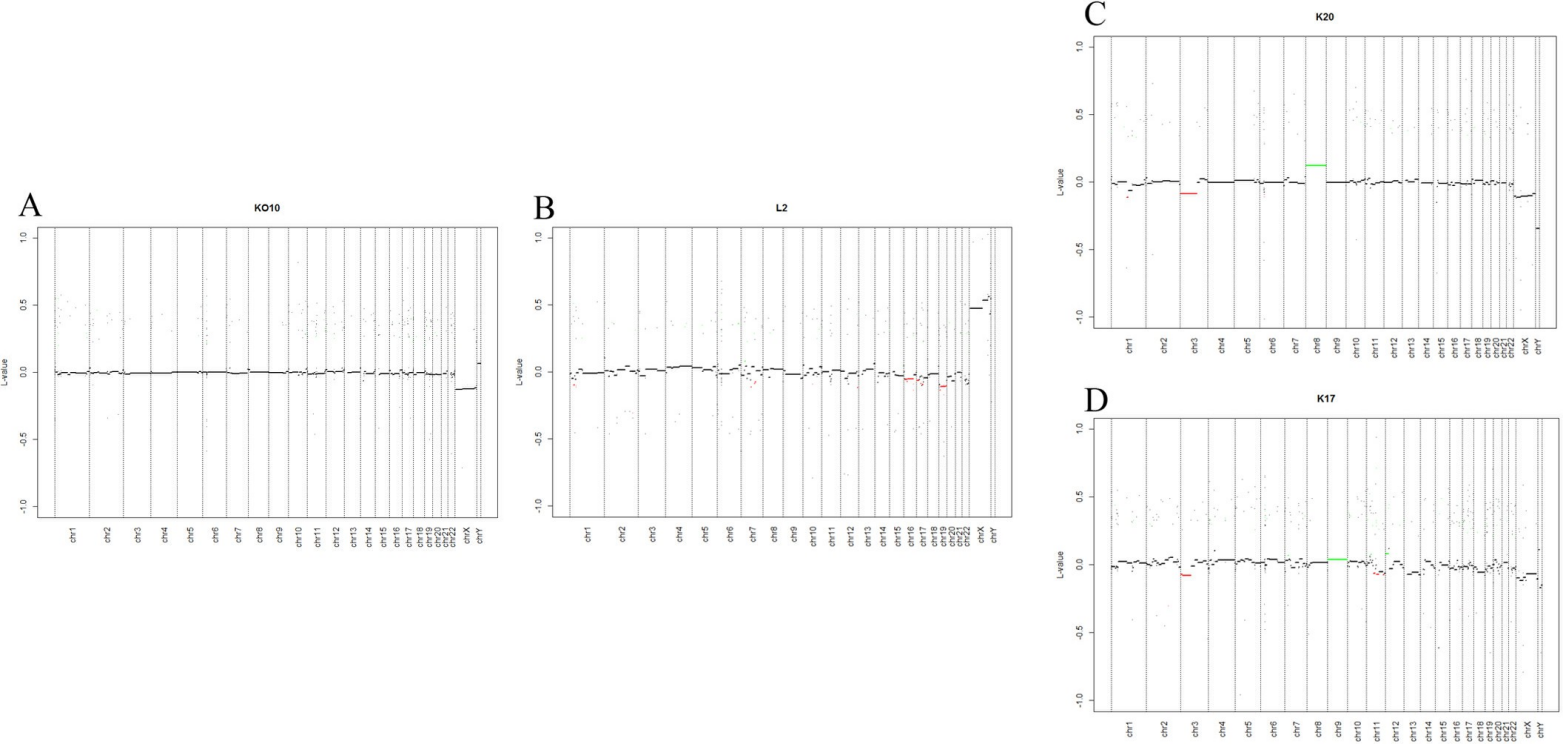
**Detailed presentation of chromosomal alterations in normal oral squamous cells (A), oral lichen planus (B) and oral squamous cell carcinoma (C, D).** For this presentation, four typical examples have been selected. The numbers below the diagrams indicate the chromosomes, green: significant chromosomal gains, red: significant chromosomal losses (as determined by *CopyNumber450k*).

Additionally, we wondered whether we could identify recurrent copy number variations in our data on OSCC or OLP samples as compared to the corresponding normal control samples. While we did not detect significant recurrent alterations in any of the OLP samples, we found recurrent genetic alterations in all OSCC samples. All analysed OSCCs displayed at least a gain of chromosomal material at chromosome 9 or a combination of gaining a region of chromosome 8 together with the simultaneous loss of genetic information at chromosome 3 (Fig 2 and S1A–S1C Fig).

Seven of the 15 cancer specimens showed a gain of genomic material in chromosome 9. Interestingly, in these seven samples (K13, K17, K23, K35, K39, K43, and K45) the alteration started at position 11,410 (as detected by the array) and ended between 6,681,608 and 141,111,396. This restricts the minimal fragment length to 6,670,198bp. The affected genomic region contains at least 39 genes, including e.g. *KANK1*, *DMRT1*, *DMRT3*, *UHRF2*, *JAK2*, *SMARCA2*, *SLC1A1*, *IL33*, *ERMP1*, and *FOXD4* (S2 Table). None of the control or OLP samples showed this gain.

Additionally, 10 of 15 OSCCs (K13, K16, K17, K20, K23, K25, K38, K39, K43, and K45), as well as one OLP (L1), showed losses at chromosome 3 starting at position 114,243 (as determined by the array). The end position ranged between 427,757 and 122,296,369. The minimal shared region contains the gene coding for *CHL1 (cell adhesion molecule L1 like)*. Several additional genomic regions at chromosome 3 and 8 showed losses commonly detected in several specimens, however, these were detected only in a minority of specimens or also found in control specimens (S2 Table).

### Identification of recurrent aberrant methylation at CpG-loci in oral lichen planus and oral squamous cell carcinoma cells

**Fig 3:**
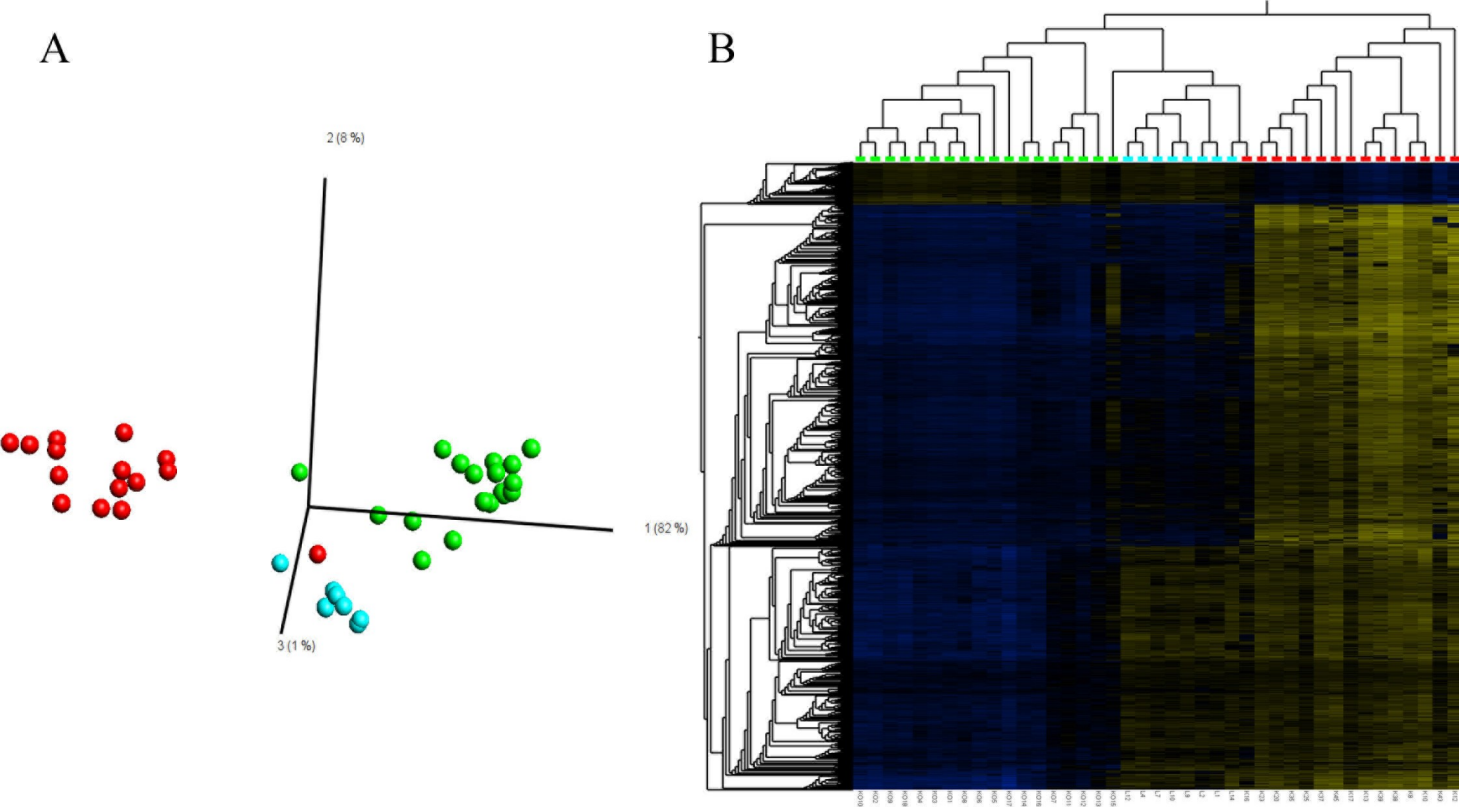
**Principal component analysis (A) and hierarchical cluster analysis (B) of OSCC (red spheres and boxes), OLP (blue) and normal controls (green) based on differentially methylated loci (FDR<1.0x10**^**-9**^**, ANOVA, n = 2192 loci).** Heatmap (B): blue: low DNA methylation, yellow: high DNA methylation. For presentation purposes, mean DNA methylation values were normalized to zero (mean = 0).

To identify differentially methylated CpG loci in OLP, OSCC, as well as healthy control tissues, an ANOVA analysis on data obtained from 450k BeadChip analysis was performed. Applying an FDR<1x10^-9^, we identified 2,192 CpG loci corresponding to 978 genes differentially methylated (S3 Table). A principal component analysis (Fig 3A) as well as a hierarchical cluster analysis (Fig 3B) based on these loci separated the normal controls from the OLP and subsequently both groups from the OSCC samples. Interestingly, DNA methylation of these CpG-loci in the distinct sample groups was clearly homogeneous. OLP samples showed an intermediate DNA methylation pattern between controls and carcinoma samples but were more similar to the former than the latter. The intermediate state of OLP between healthy tissue and OSCC is also supported by further analyses applying a t-test statistic to compare controls and OSCC (S3 Fig). In a principal component analysis based on 2,548 loci distinguishing controls and OSCC (FDR<1.08x10^-10^), OLP samples located mainly between controls and OSCC samples. The same holds true in a PCA of the 7,611 CpG loci showing the highest variance in the data set (σ/σ_max_≥0.4) as identified by an unsupervised data analysis approach (S4 Fig). Two samples (a single carcinoma and a single control sample) clustered with OLP samples. This might be due to tissue heterogeneity and different compartments within these samples.

### Localisation of differentially methylated CpG loci in regions of distinct chromosomal states

**Fig 4:**
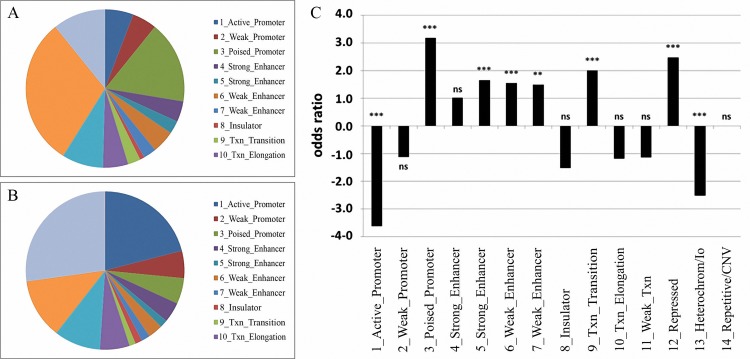
Enrichment of differentially methylated loci located in distinct chromosomal states. To determine the individual chromosomal state, data provided by the Ensembl database on the chromosomal state in NHEK cells were used. Pie charts of the chromatin states of differentially methylated loci (FDR<1.0x10^-9^, ANOVA, n = 2192 loci) (A) and all loci present on the HumanMethylation450 BeadChip included into this study (B) are presented. (C) Bar chart showing odds ratios of selected chromosomal states affected by differential DNA methylation in OSCC, OLP, and controls. ***: p<0.001; **: p<0.01; *: p<0.05; ns: not significant.

To get further insight into the localisation of differentially methylated CpG-loci in chromosomal regions with distinct chromatin states, we used data on the chromosomal state in NHEK cells available from the Ensembl database. While the identified regions containing differentially methylated loci were depleted of active promoters (3.62fold, p<0.0001) as well as heterochromatic regions (2.52fold, p<0.0001) in NHEK cells, we found a significant enrichment of differentially methylated loci in repressed chromatin (2.47fold, p<0.0001), transcription transition (2.00fold, p<0.0001), enhancer (1.5fold, p<0.0001) and in particular in poised promoter (3.18fold, p<0.0001) sequences of NHEK cells (Fig 4). Furthermore, it turned out that the aberrantly methylated loci were also enriched for loci located in CpG-islands, DNase I hypersensitive sites, enhancers and known differentially methylated regions (S2 Fig).

### Gene ontology analysis

A gene ontology analysis of the genes corresponding to the 2,192 different loci using the GOrilla tool revealed 117 significantly enriched processes (FDR<0.01). Of these 39 processes were enriched ≥2 fold (S4 Table). Nine GO terms were enriched ≥4 fold: glandular epithelial cell differentiation (6.79 fold), signal transduction involved in regulation of gene expression (6.54 fold), proximal/distal pattern formation (6.23 fold), forelimb morphogenesis (5.75 fold), embryonic forelimb morphogenesis (5.66 fold), cell differentiation in spinal cord (4.67 fold), cell fate specification (4.52 fold), dorsal/ventral pattern formation (4.22 fold) and peptide hormone secretion (4.00 fold). Overall this list contains processes involved e.g. in cell differentiation, embryonic development, and signal transduction.

### Oral lichen planus as a putative precursor lesion of oral squamous cell carcinoma

In a follow-up study with 327 patients, the annual malignant transformation rate amounted to about 0.5% [43] and OLP has been considered a putative precursor lesion of OSCC. Therefore we tested the hypothesis that genes becoming aberrantly methylated in the (putative) first step, i.e. development of OLP from healthy tissue, and those genes which become aberrantly methylated during the putative second step, i.e. progression of OLP to OSCC, show distinct characteristics. Our approach is similar to the one previously described in hepatocellular carcinoma [44].

**Fig 5:**
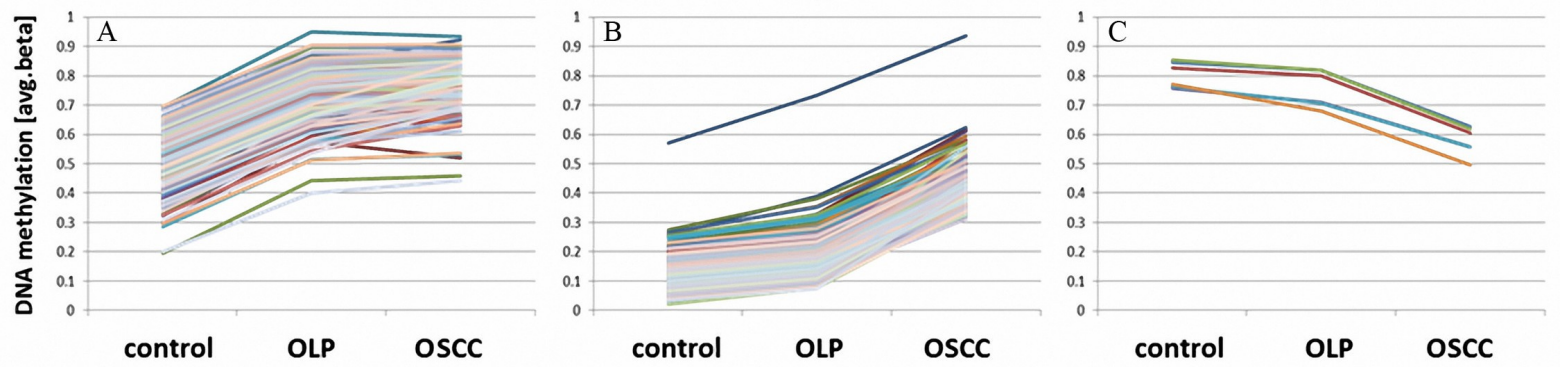
DNA methylation values of loci assumed to be hypermethylated in both oral lichen planus (OLP) and oral squamous cell carcinoma (OSCC) as compared to normal controls. Methylation gained from normal control to OLP (A), loci exclusively hypermethylated in oral squamous cell carcinoma (OSCC) (gaining methylation in OSCC; B) and loci continuously losing DNA methylation from controls via OLP to OSCC (C). Different colors of lines indicate individual CpG loci.

**Fig 6:**
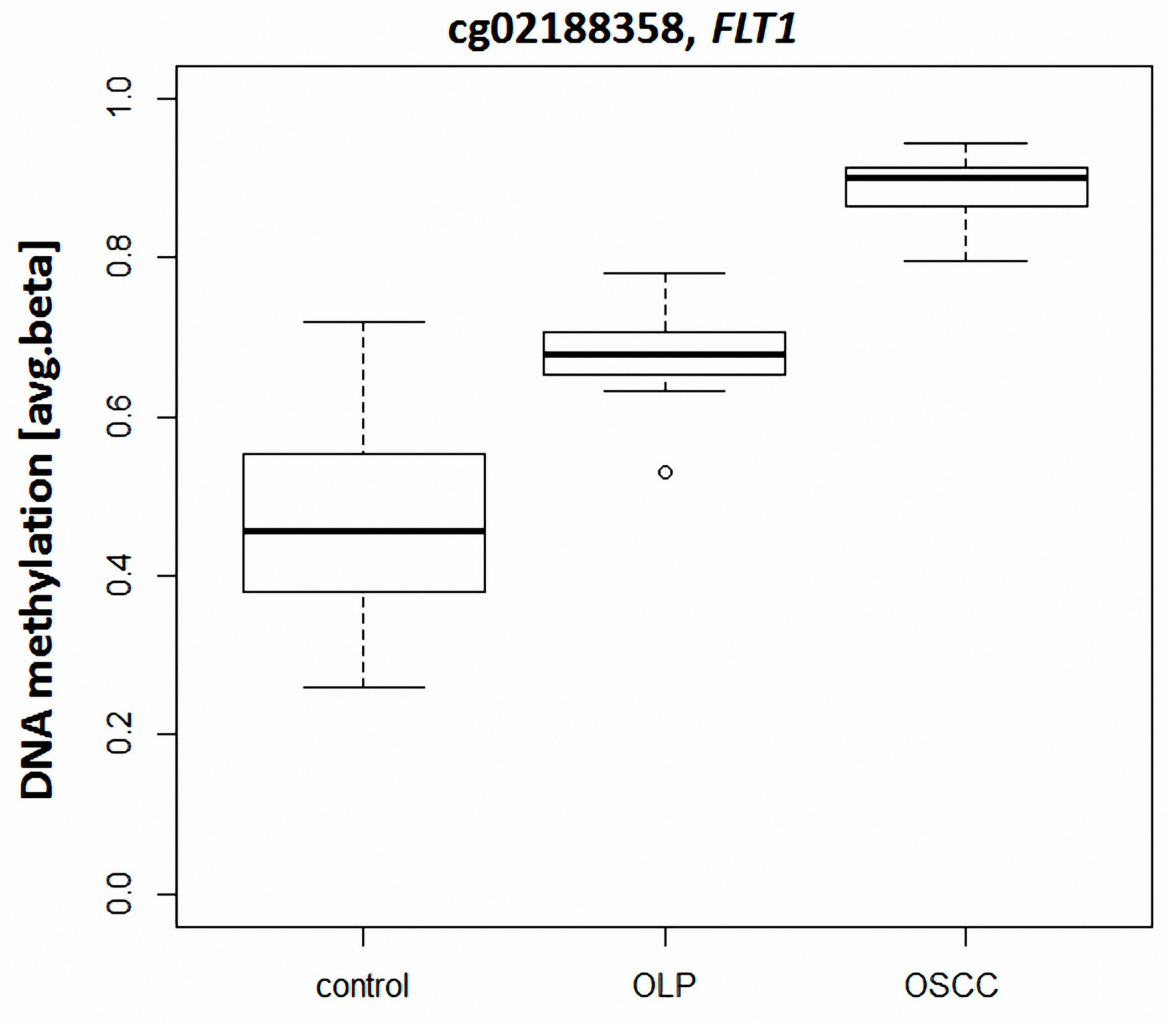
Boxplot indicating the DNA methylation value of the CpG loci cg02188358 (allocated to the gene FLT1). This locus shows a continuous significant increase in DNA methylation from control tissue to oral lichen planus (OLP) to oral squamous cell carcinoma (OSCC).

We classified the 2,192 differentially methylated loci identified above into i) those hypermethylated in both OLP and OSCC, as compared to the control samples (n = 196; OLP and OSCC vs. control), ii) loci hypermethylated (n = 634; OLP and control vs. OSCC) or iii) loci hypomethylated (n = 6; OLP and control vs. OSCC) exclusively in OSCC (Fig 5). For this approach, only loci showing a difference of the means (delta.beta) greater than 0.2 between the two groups compared were considered as differentially methylated. 1,356 loci did not meet this criterion and were therefore excluded from this analysis; no locus was found to be hypomethylated in both OSCC and OLP, as compared to controls. One locus (cg02188358, *FLT1*) showed hypermethylation in OLP as compared to control samples (delta.beta(OLP-control)>0.2), as well as in OSCC as compared to OLP (delta.beta(OSCC-OLP)>0.2)(p<0.001; Fig 6). Loci hypermethylated in OLP and OSCC were significantly depleted of loci located in active promoters (OR: 0.20, p<0.0001) and regions of heterochromatin in NHEK cells (OR: 0.34, p<0.0001). In contrast, this group was enriched for loci located in poised promoters (OR: 2.73, p<0.0001), weak enhancers (OR: 1.89, p<0.05) and repressed chromatin (OR: 2.73, p<0.0001). Similarly, the set of CpG loci hypermethylated exclusively in OSCC was also enriched for loci located in poised promoters (OR: 2.82, p<0.0001), weak enhancers (OR: 1.56, q<0.05) as well as repressed genomic sequences (OR: 2.51, p<0.0001) in NHEK cells, whereas it was depleted for loci located in active promoters (OR: 0.29, p<0.0001) and heterochromatic regions (OR: 0.42, p<0.0001).

### Epigenetic age of oral lichen planus and oral squamous cell carcinoma samples

Because aging is correlated with distinct changes in the DNA methylome, DNA methylation can be used to calculate the (biological) age of a sample [45] and deviations of the epigenetic age from the chronological age have been associated with numerous diseases, including several cancer entities [46]. Therefore we used the DNAm age (DNA Methylation Age) calculator to determine the epigenetic age of the OSCC, OLP, as well as control samples (S5 Fig). However, we found no marked acceleration or deceleration of aging, neither in normal oral tissue samples nor in OLP or OSCC.

## Discussion

By assaying more than 450,000 CpG sites in the genome, we studied the patterns of altered methylation between normal oral mucosa, OLP, and OSCC. In this analysis, distinct patterns of altered methylation arise that may contribute to a better understanding of OSCC and its carcinogenesis.

The aetiology of OLP and its potential to represent a premalignant phenotype remain poorly understood [30, 32, 47]. However, there is strong evidence concerning the relationship between inflammation and tumour development. It is obvious that the oral cavity with its special environmental conditions, e.g. bacterial and viral infections and continuing chemical irritations, is of particular risk to develop chronic inflammatory stress. It is known that the interplay of inflammatory microenvironment and genetic and epigenetic alterations can lead to the development of various types of cancer over time [48, 49]. Moreover, it has been indicated that epigenetic changes occurring in cancer, e.g. increased DNA methylation and gene silencing are accelerated in inflamed tissue [50].

This study aimed to identify genetic and epigenetic changes found in both OLP and in OSCC, as well as alterations unique to OSCC. This information might help us in defining molecular steps in the development of OLP and the subsequent onset of OSCC [44]. While we identified several genetic alterations in OLP none of these were restricted to OLP as they could also be found in normal controls. Only two individual OLP samples showed similarities in their alteration pattern. Thus, at least in the limited number of OLP samples included in this study, no genetic alteration specific for OLP, which might allow differentiation from normal tissue samples, could be identified. Furthermore, the groups of OLP, OSCC and control patients differed in their median age. The healthy control group consisted mainly of rather young people who underwent elective wisdom tooth removal or orthognathic surgery; the development of OLP and especially OSCC occurs at a more advanced age. A greater age means longer exposure to environmental factors and possibly a different lifestyle, which could influence methylation patterns and accelerate cancer risk [51]. For these reasons a larger study with matched groups could reduce related confounders. In contrast, genetic alterations characteristic for OSCC have been found on the chromosomes 3, 8 and 9. Sequences recurrently gained on chromosome 9 include the genes encoding the KN motif and ankyrin repeat domains 1 (*KANK1*), involved in induction of apoptosis [52, 53], as well as known key players of carcinogenesis in gastrointestinal and haematological neoplasia like UHRF2 protein ligase (*ubiquitin-like with PHD and ring finger domains 2*), JAK2 (*Janus kinase 2*) or SMARCA2 (*SWI/SNF related*, *matrix associated*, *actin dependent regulator of chromatin*, *subfamily a*, *member 2*) [54–59]. While these gained sequences on chromosome 9 were found in 47% of OSCC, none of the control or OLP samples carried them. Furthermore, 67% of all OSCCs showed sequence losses at chromosome 3, starting at position 114,243. The minimal region lost in these samples carried only the gene encoding for CHL1 (*cell adhesion molecule L1 like*). The encoded protein may be involved in signal transduction pathways. Alterations of CHL1 have been correlated with several tumour entities and have been suggested to function as tumour biomarkers e.g. in breast cancer [60–63]. Interestingly, only one OLP case also showed this specific alteration, which might possibly argue for an advanced premalignant state of this particular sample. Sequence gains were also identified on chromosome 8 in several patients; however, these were not highly recurrent. Overall, we identified recurrent genetic alterations in OSCC but not in OLP. Number and size of the genetic alterations found in OSCC were significantly increased as compared to OLP and normal controls, arguing for increased genetic instability in the neoplastic samples. Since no change in number and size of genetic alteration could be detected when comparing OLP and controls, our data argue that genetic stability is not significantly impaired in OLP.

In contrast to the genetic alterations, we identified numerous alterations in the DNA methylation pattern of both OLP and OSCC, as compared to the control tissues. DNA methylome data clearly distinguished all three sample groups. Interestingly, a large number of CpG loci aberrantly methylated in OLP were found equally methylated in OSCC. This supports the hypothesis that OLP is a precursor lesion of OSCC; parts of the DNA methylome are already altered and thus may prepare the affected (precancerous) cell to transform into a cancer cell. However, numerous additional epimutations were uniquely characteristic of OSCC. This holds particularly true for the hypermethylation of loci located in bivalent promoters. Interestingly, similar findings have been reported in liver diseases (fatty liver disease, liver cirrhosis and hepatocellular carcinoma (HCC)). Here, increasing numbers of epimutations were reported during disease progression, while hypermethylation of bivalent promoters and polycomb repressor complex 2 (*PRC2*) target genes was restricted to HCC [44, 64]. In line with our findings, hypermethylation of bivalent promoters has been described as a general characteristic of malignant tumours [65].

In summary, the results of our study support the hypothesis that OLP is a precursor lesion of OSCC. While OLP and OSCC already share numerous epigenetic alterations, further epimutations, as well as genetic alterations, are characteristic for OSCC. Our study findings might help to improve early diagnosis, to identify patients at risk for the development of OSCC and to ensure appropriate patient management.

## Supporting information

S1 Table Detailed overview of the samples included in the DNA methylation analyses. (XLSX)

S2 Table Detailed overview of chromosomal alterations of the samples included in the DNA methylation analyses. (XLSX)

S3 Table Detailed overview of methylated CpG-loci of the samples included in the DNA methylation analyses. (XLSX)

S4 Table Detailed overview of the 117 significantly enriched processes of the gene ontology analysis. (XLSX)

S1 Fig Detailed presentation of gains and losses of genomic regions in all individual samples included in this study. The numbers below of the diagrams indicate the chromosomes, green: significant chromosomal gains, red: significant chromosomal losses (as determined by *CopyNumber450k*). Data from oral squamous cell carcinoma (A), oral lichen planus (B) and normal controls (C) are presented.
(TIF)

S2 Fig Enrichment of differentially methylated loci located in distinct chromosomal states. To determine the individual chromosomal state data provided by the allocation of individual loci present on the HumanMethylation450 BeadChip provided by Illumina was used. The bar chart shows fold enrichment of selected chromosomal states affected by differential DNA methylation in OSCC, OLP, and controls. ***: p<0.001; **: p<0.01; *: p<0.05; ns: not significant.
(TIF)

S3 Fig **Hierarchical cluster analysis (A) and principal component analysis (B) based on the DNA methylation values of 2,548 CpG loci differentiating control samples (green spheres and green boxes) and oral squamous cell carcinoma (red spheres and red boxes) (FDR<1.08x10**^**-10**^**, t-test).** Heatmap (A): blue: low DNA methylation, yellow: high DNA methylation. For presentation purposes, mean DNA methylation values were normalized to zero (mean = 0). PCA: blue spheres indicate oral lichen planus samples (position of OLP in PCA is shown, OLP data was not included in t-test statistic to determine loci differentially methylated between controls and OSCC).
(TIF)

S4 Fig Principal component analysis of 7,611 CpG with high variance in the data set (unsupervised approach, σ/σ_max_≥0.4). Green spheres: control samples, blue: oral lichen planus (OLP) samples, red: oral squamous cell carcinoma (OSCC).
(TIF)

S5 Fig Scatterplot demonstrating the correlation of the chronological age of the patient with the epigenetic age (“methylation age”) as determined by Horvath’s age calculator. Blue dots: control samples, green: oral lichen planus, red: oral squamous cell carcinoma. The black line indicates positions with chronological age = methylation age.
(TIF)
