# Supplementary figures and images for: Recurrent chromosomal and epigenetic alterations in oral squamous cell carcinoma and its putative premalignant condition oral lichen planus
Source: PLoS One. 2019 Apr 9;14(4):e0215055. doi: 10.1371/journal.pone.0215055 (PMC6456184; doi:10.1371/journal.pone.0215055)

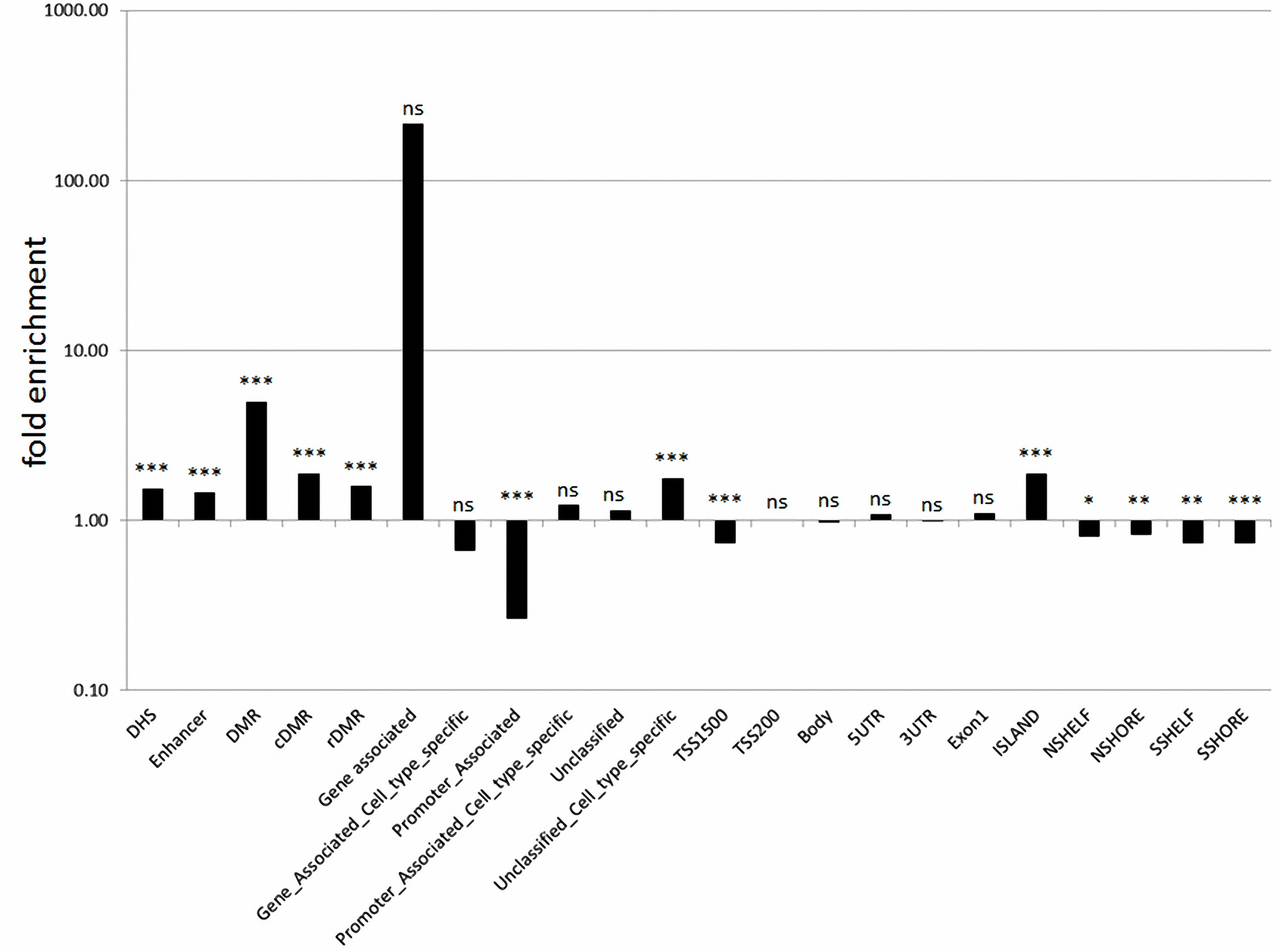

Supplement: S2 Fig — To determine the individual chromosomal state data provided by the allocation of individual loci present on the HumanMethylation450 BeadChip provided by Illumina was used. The bar chart shows fold enrichment of selected chromosomal states affected by differential DNA methylation in OSCC, OLP, and controls. ***: p<0.001; **: p<0.01; *: p<0.05; ns: not significant. (TIF) [file pone.0215055.s006.tif]

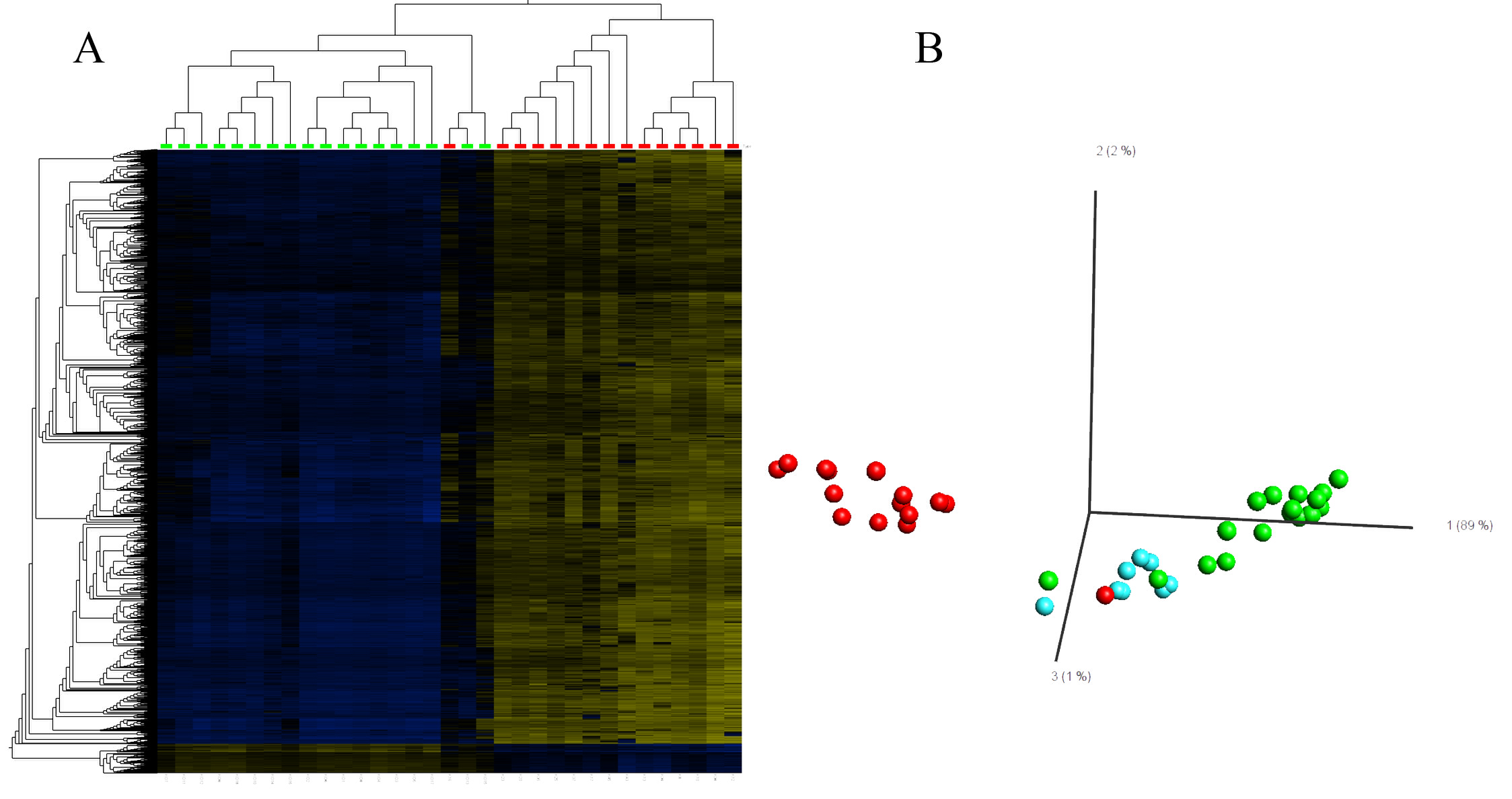

Supplement: S3 Fig — Hierarchical cluster analysis (A) and principal component analysis (B) based on the DNA methylation values of 2,548 CpG loci differentiating control samples (green spheres and green boxes) and oral squamous cell carcinoma (red spheres and red boxes) (FDR<1.08x10^-10^, t-test). Heatmap (A): blue: low DNA methylation, yellow: high DNA methylation. For presentation purposes, mean DNA methylation values were normalized to zero (mean = 0). PCA: blue spheres indicate oral lichen planus samples (position of OLP in PCA is shown, OLP data was not included in t-test statistic to determine loci differentially methylated between controls and OSCC). (TIF) [file pone.0215055.s007.tif]

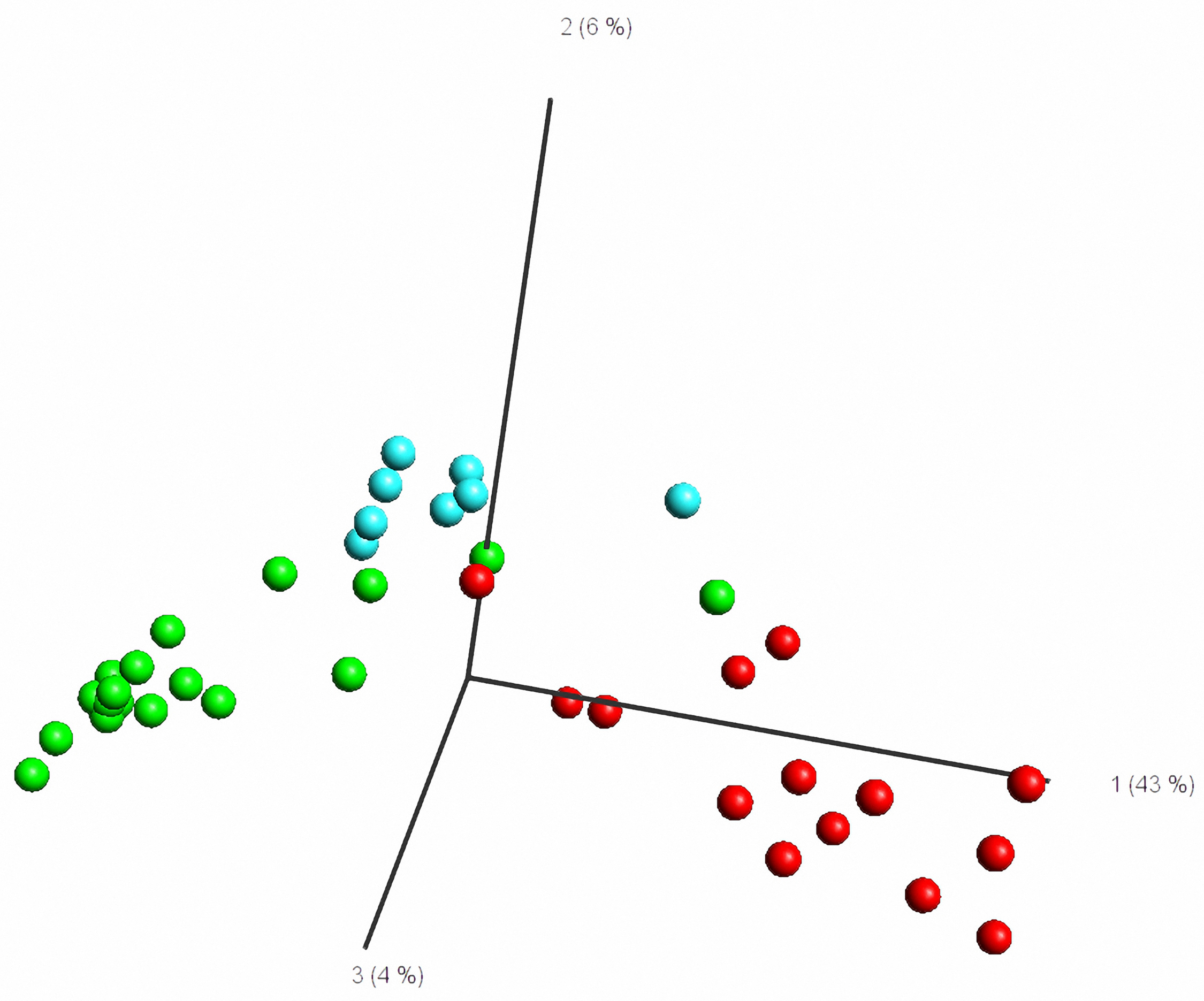

Supplement: S4 Fig — Green spheres: control samples, blue: oral lichen planus (OLP) samples, red: oral squamous cell carcinoma (OSCC). (TIF) [file pone.0215055.s008.tif]

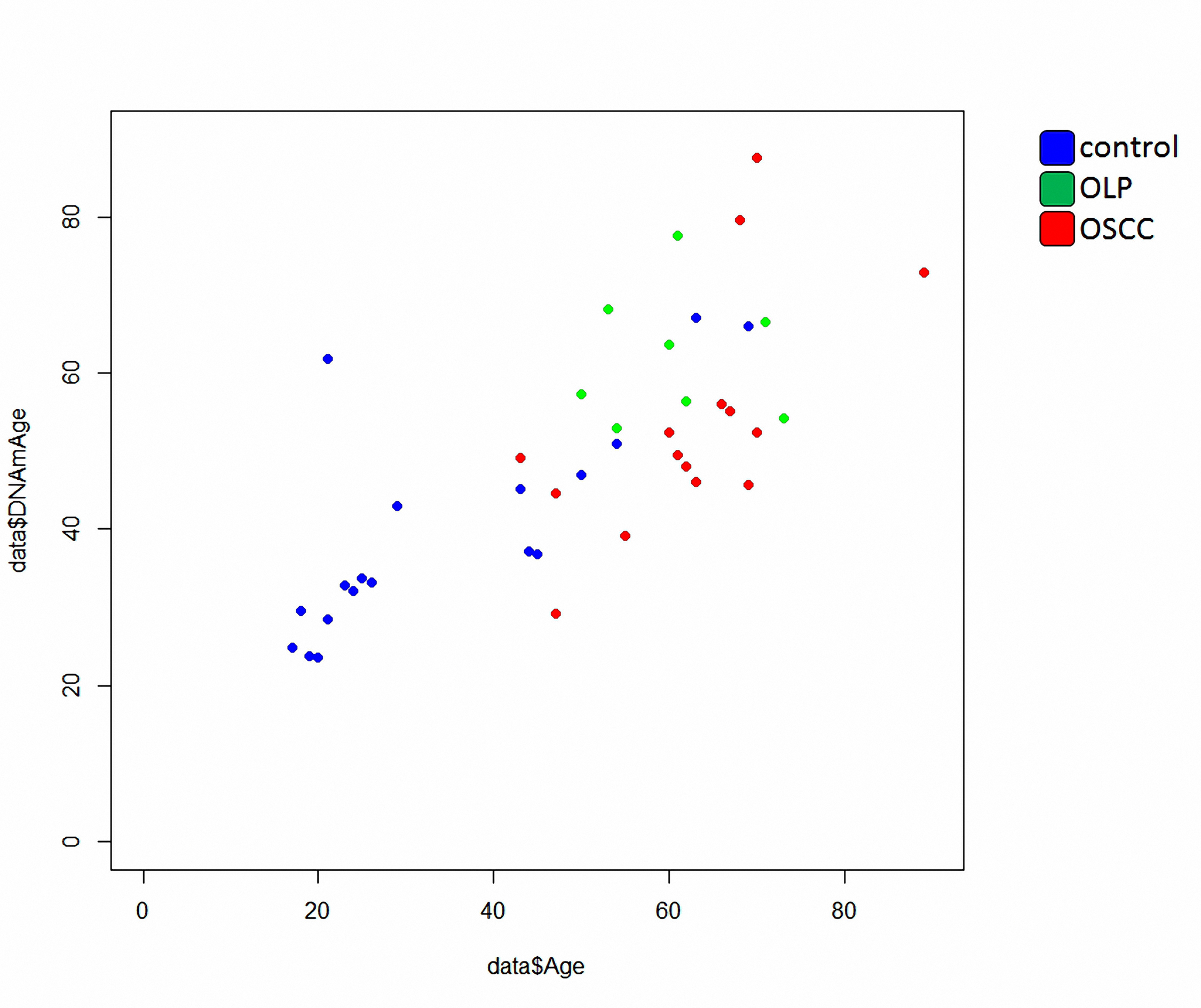

Supplement: S5 Fig — Blue dots: control samples, green: oral lichen planus, red: oral squamous cell carcinoma. The black line indicates positions with chronological age = methylation age. (TIF) [file pone.0215055.s009.tif]
